# Supplementary material for: Dynamics of the formation of flat clathrin lattices in response to growth factor stimulus
Source: PLoS Comput Biol. 2026 Mar 11;22(3):e1014013. doi: 10.1371/journal.pcbi.1014013 (PMC13012621; doi:10.1371/journal.pcbi.1014013)
Supplement: S1 Text — (PDF) [file pcbi.1014013.s001.pdf]

# Supporting information for Dynamics of the formation of flat clathrin lattices in response to growth factor stimulus

Lingxia Qiao[1,2], Marco A. Alfonzo-Méndez[3], Justin W. Taraska[3], and Padmini Rangamani*[2, 4]

[1]School of Data Science, Fudan University, Shanghai, China
[2]Department of Pharmacology, School of Medicine, University of California San Diego, San Diego, California,United States of America.
[3]Biochemistry and Biophysics Center, National Heart, Lung, and Blood Institute, National Institutes of Health,Bethesda, Maryland, United States of America.
[4]Department of Mechanical and Aerospace Engineering, Jacob's School of Engineering, University of California San Diego, San Diego, California, United States of America.

*To whom correspondence must be addressed: prangamani@health.ucsd.edu

## 1 The Turing model cannot simultaneously achieve the increase of cluster size and the increase of cluster number

Motivated by the Turing model in [1], we used the following equations to describe the dynamics of AP-2 and clathrin on the cell membrane (panel A in S8 Fig):

$$\begin{aligned}\frac{\partial[AP\text{-}2]}{\partial t} =& -\beta\Big([AP\text{-}2] - \frac{N\text{-}[AP\text{-}2]-[Clat]}{N-[AP\text{-}2]_{ss}-[Clat]_{ss}}[AP\text{-}2]_{ss}\Big)\\ &+\mu\frac{N-[AP\text{-}2]-[Clat]}{N-[AP\text{-}2]_{ss}-[Clat]_{ss}}\frac{[AP\text{-}2]}{[AP\text{-}2]_{ss}}\Big([AP\text{-}2]-[AP\text{-}2]_{ss}\Big)+D_{AP\text{-}2}\triangle[AP\text{-}2] \qquad \text{(S1)}\end{aligned}$$

$$\begin{aligned}\frac{\partial[Clat]}{\partial t} =& -b\Big([Clat] - \frac{[AP\text{-}2]}{[AP\text{-}2]_{ss}}\frac{N-[AP\text{-}2]-[Clat]}{N-[AP\text{-}2]_{ss}-[Clat]_{ss}}[Clat]_{ss}\Big)\\ &-m_1\frac{N-[AP\text{-}2]-[Clat]}{N-[AP\text{-}2]_{ss}-[Clat]_{ss}}\Big([Clat]-[Clat]_{ss}\Big)\\ &+m_2\frac{N-[AP\text{-}2]-[Clat]}{N-[AP\text{-}2]_{ss}-[Clat]_{ss}}\frac{[Clat]}{[Clat]_{ss}}\Big([AP\text{-}2]-[AP\text{-}2]_{ss}\Big)+\widetilde{D_{Clat}}\triangle[Clat] \qquad \text{(S2)}\end{aligned}$$

where $[AP\text{-}2]$ and $[Clat]$ denote the concentrations of AP-2 and clathrin on the cell membrane, respectively. All terms in Equations (S1) and (S2) are the same as those in [1] except the diffusion terms. The diffusion terms $D_{AP-2}\triangle[AP\text{-}2]$ and $\widetilde{D_{Clat}}\triangle[Clat]$ in Equations (S1) and (S2) are simpler than that in [1], but the Turing pattern still can be maintained. Four mechanisms that are key to Turing pattern formation are as follows: 1) the enhanced recruitment of AP-2 if one AP-2 has already bound to the cell membrane (the curved arrow in the panel A in S8 Fig); 2) the recruitment of clathrin to the cell membrane caused by AP-2 (the arrow from AP-2 to clathrin in the panel A in

S8 Fig); 3) the steric repulsion between clathrin and AP-2 (the arrow from clathrin to AP-2 in the panel A in S8 Fig); 4) much slower diffusion coefficient of AP-2 compared to that of clathrin. In the first two mechanisms, the reaction strength is denoted by $\mu$ and $m_2$. For the third mechanism, it is achieved by multiplying $\frac{N-[AP\text{-}2]-[Clat]}{N-[AP\text{-}2]_{ss}-[Clat]_{ss}}$ to each reaction term: the larger the $[AP\text{-}2]+[Clat]$ is, the smaller the $\frac{N-[AP\text{-}2]-[Clat]}{N-[AP\text{-}2]_{ss}-[Clat]_{ss}}$ is, leading to less recruitment events. Here, $N$ is set to be a large enough number to ensure $[AP\text{-}2]+[Clat] \leq N$ hold all the time, and the subscript $ss$ denotes the homogeneous steady-state value. As for the fourth mechanism, we set the ratio of diffusion coefficients between AP-2 and clathrin $D_{AP\text{-}2}/\widetilde{D_{Clat}}$ as 0.0377 in our simulations. Note that the value 0.0377 was chosen because it satisfies the fourth mechanism, but it may not be realistic for the clathrin-AP-2 system.

The values of kinetic parameters in Equations (S1) and (S2) are listed in S3 Table, which are determined by searching previous clathrin models or the requirement of Turing instability. According to the clathrin model in [2], the dissociation rate of AP-2 and $PIP_2$ is 1 $s^{-1}$, and the dissociation rate of clathrin and AP-2 is 0.03 $s^{-1}$. Therefore, we set the dissociation rate of AP-2 and cell membrane $\beta$ to be 1 $s^{-1}$, and the dissociation rate of clathrin and cell membrane $b$ 0.03 $s^{-1}$. Besides, the values of $N$, $[AP\text{-}2]_{ss}$ and $[Clat]_{ss}$ were chosen to match the scale of AP-2 in [2] ($\sim$ 361 copies/µm$^2$). Other kinetic parameters were determined by the constraints of Turing instability, such as $\mu$, $m_1$, $m_2$, $D_{AP-2}$ and $\widetilde{D_{Clat}}$. It should be noted that the value of $D_{AP-2}$ is 1% of AP-2 translational diffusion constant on the membrane in [2], and that the value of $\widetilde{D_{Clat}}$ is also 1% of clathrin translational diffusion constant in [2]. This rescaling of the diffusion constant ensures the correct size of each clathrin cluster.

We first tested whether the Turing model can capture the experimental observations of FCL dynamics after the stimulus of EGF. By simulating the Turing model, we obtained a stable pattern, corresponding to the system without stimulus (the first plot in the panel B in S8 FigB). Then, we increased the association rate of AP-2 and membrane $\mu$ by 80% to mimic the effect of adding EGF. Under this change, the system experiences two stages: (1) in [0, 15 seconds], each clathrin cluster grows in size but no new clusters emerge (panel B in S8 Fig); (2) after 15 seconds, each large clathrin cluster disassembles into small clusters, leading to the increase in the number of clusters and decrease in the size of each cluster. To make the trend of cluster size and cluster number more clear, we used the image processing tool to obtain the boundary of each cluster and then calculated the cluster size and cluster number quantitatively (panel C in S8 Fig). It can be seen that the increase in cluster size and the increase in total cluster number occur at different time intervals, i.e., [0 15 seconds] and after 15 seconds, respectively. However, as we can see from the experimental data (Fig 1E), the increase in the size and that in the number happens simultaneously. AP-2 is co-localized with clathrin (Fig 1D) and exhibits similar dynamics to clathrin. Taken together, the Turing model cannot capture the FLC dynamics under the EGF stimulus.

# References


[1] Haselwandter, C. A., Calamai, M., Kardar, M., Triller, A. & Azeredo da Silveira, R. Formation and stability of synaptic receptor domains. Phys. Rev. Lett. **106**, 238104 (2011). URL `https://link.aps.org/doi/10.1103/PhysRevLett.106.238104`.

[2] Guo, S.-K., Sodt, A. J. & Johnson, M. E. Large self-assembled clathrin lattices spontaneously disassemble without sufficient adaptor proteins. PLOS Computational Biology **18**,

1–24 (2022). URL https://doi.org/10.1371/journal.pcbi.1009969.
