# Supplementary material for: Dynamics of the formation of flat clathrin lattices in response to growth factor stimulus
Source: PLoS Comput Biol. 2026 Mar 11;22(3):e1014013. doi: 10.1371/journal.pcbi.1014013 (PMC13012621; doi:10.1371/journal.pcbi.1014013)
Supplement: S1 Table — (PDF) [file pcbi.1014013.s002.pdf]

**Parameters**	**Explanations**
Clathrin number	300
AP-2-clathrin binding rate $k_{AP\text{-}2 \cdot Clat}$	0.0012 $\mu M^{-1} s^{-1}$
Clathrin-clathrin binding rate without AP-2 $k_{Clat \cdot Clat}$	0
AP-2 translational diffusion diffusion coefficient in x, y, and z directions: $D_{AP\text{-}2,x}, D_{AP\text{-}2,y}, D_{AP\text{-}2,z}$	$D_{AP\text{-}2,x} = D_{AP\text{-}2,y} = 0.5\ \mu m^2/s$, $D_{AP\text{-}2,z} = 0$
AP-2 rotational diffusion diffusion coefficient in x, y, and z directions: $D_{R,AP\text{-}2,x}, D_{R,AP\text{-}2,y}, D_{R,AP\text{-}2,z}$	$D_{R,AP\text{-}2,x} = D_{R,AP\text{-}2,y} = 0$, $D_{R,AP\text{-}2,z} = 0.01\ rad^2/\mu s$
Clathrin rotational diffusion coefficient in x, y, and z directions: $D_{R,Clat,x}, D_{R,Clat,y}, D_{R,Clat,z}$	$D_{R,Clat,x} = D_{R,Clat,y} = D_{R,Clat,z}$ $= D_{R,Clat} = 0.03\ rad^2/\mu s$
**Parameters used in Fig 2**	
AP-2-clathrin dissociation rate $d_{AP\text{-}2 \cdot Clat}$	0.003 s^{-1}
Clathrin-clathrin binding rate with AP-2 $k_{(AP\text{-}2 \cdot)Clat \cdot Clat}$	$20 \times 0.913\ \mu M^{-1} s^{-1}$
Clathrin translational diffusion coefficient in x, y, and z directions: $D_{Clat,x}, D_{Clat,y}, D_{Clat,z}$	$D_{Clat,x} = D_{Clat,y} = D_{Clat,z}$ $= D_{Clat} = 13\ \mu m^2/s$
AP-2 number	150
Clathrin-clathrin dissociation rate $d_{Clat \cdot Clat}$	10 s^{-1}
**Parameters used in Fig 3**	
$d_{AP\text{-}2 \cdot Clat}$	0.003 s^{-1}
$k_{(AP\text{-}2 \cdot)Clat \cdot Clat}$	$50 \times 0.913\ \mu M^{-1} s^{-1}$
D_{Clat}	13 $\mu m^2/s$
AP-2 number	[10, 50, 100, 150, 200, 250, 300, 350, 400]
$d_{Clat \cdot Clat}$	10 s^{-1}
**Parameters used in Fig 4**	
$d_{AP\text{-}2 \cdot Clat}$	0.003 s^{-1}
$k_{(AP\text{-}2 \cdot)Clat \cdot Clat}$	$[1, 25, 50, 75, 100] \times 0.913\ \mu M^{-1} s^{-1}$
D_{Clat}	13 $\mu m^2/s$
AP-2 number	100
$d_{Clat \cdot Clat}$	10 s^{-1}
**Parameters used in Fig 5**	
$d_{AP\text{-}2 \cdot Clat}$	0.003 s^{-1}
$k_{(AP\text{-}2 \cdot)Clat \cdot Clat}$	$50 \times 0.913\ \mu M^{-1} s^{-1}$
D_{Clat}	$[1, 1/5, 1/10, 1/20, 1/30, 1/40] \times 13\ \mu m^2/s$
AP-2 number	100
$d_{Clat \cdot Clat}$	10 s^{-1}
**Parameters used in Fig 6A**	
$d_{AP\text{-}2 \cdot Clat}$	0.0003 s^{-1}
$k_{(AP\text{-}2 \cdot)Clat \cdot Clat}$	$0.913\ \mu M^{-1} s^{-1}$

D_{Clat}	$13\ \mu m^2/s$
AP-2 number	100
$d_{Clat \cdot Clat}$	10 s^{-1}
**Parameters used in S3 Fig**	
$d_{AP\text{-}2 \cdot Clat}$	0.0003 s^{-1}
$k_{(AP\text{-}2 \cdot)Clat \cdot Clat}$	$50 \times 0.913\ \mu M^{-1} s^{-1}$
D_{Clat}	$13\ \mu m^2/s$
AP-2 number	100
$d_{Clat \cdot Clat}$	10 s^{-1}
$k_{AP-2 \cdot Clat}$	$0.5 \times 0.0012\ \mu M^{-1} s^{-1}$ in panel A<br>$20 \times 0.0012\ \mu M^{-1} s^{-1}$ in panel B
