# Supplementary material for: Dynamics of the formation of flat clathrin lattices in response to growth factor stimulus
Source: PLoS Comput Biol. 2026 Mar 11;22(3):e1014013. doi: 10.1371/journal.pcbi.1014013 (PMC13012621; doi:10.1371/journal.pcbi.1014013)
Supplement: S2 Table — (PDF) [file pcbi.1014013.s003.pdf]

| | |
|---|---|
| First binding site to clathrin | $(3, 3\sqrt{3}, 0)$ |
| Second binding site to clathrin | $(3, -3\sqrt{3}, 0)$ |
| Third binding site to clathrin | $(-6, 0, 0)$ |
| Binding site to AP-2 | $(0, 0, -3)$ |
