# Supplementary material for: Dynamics of the formation of flat clathrin lattices in response to growth factor stimulus
Source: PLoS Comput Biol. 2026 Mar 11;22(3):e1014013. doi: 10.1371/journal.pcbi.1014013 (PMC13012621; doi:10.1371/journal.pcbi.1014013)
Supplement: S3 Table — (PDF) [file pcbi.1014013.s004.pdf]

Parameters	Explanations	Values	Ref.
**Parameters used in the Turing model**			
β	AP-2 & membrane dissociation rate	1 s^{-1}	[2]
μ	AP-2 & membrane association rate	1.4 s^{-1}	Fitted
b	Clathrin & membrane dissociation rate	0.03 s^{-1}	[2]
m_1	Clathrin & membrane dissociation rate caused by unspecified molecules	0.8s^{-1}	Fitted
m_2	Recruitment rate of clathrin by AP-2	10 s^{-1}	Fitted
D_{AP-2}	Diffusion coefficient of AP-2	0.049 µm^{2}s^{-1}	1/100 of the value in [2]
$\widetilde{D_{Clat}}$	Diffusion coefficient of clathrin	1.3 µm^{2}s^{-1}	1/100 of the value in [2]
N	Maximal total concentration	500 µm^{-2}	Same scale as in [2]
$[AP\text{-}2]_{ss}$	Homogeneous steady-state value of [AP-2]	25 µm^{-2}	Same scale as in [2]
$[Clat]_{ss}$	Homogeneous steady-state value of [Clat]	25 µm^{-2}	Same scale as in [2]
