# Supplementary figures and images for: Dynamics of the formation of flat clathrin lattices in response to growth factor stimulus
Source: PLoS Comput Biol. 2026 Mar 11;22(3):e1014013. doi: 10.1371/journal.pcbi.1014013 (PMC13012621; doi:10.1371/journal.pcbi.1014013)

**A** Sochacki et al., 2021

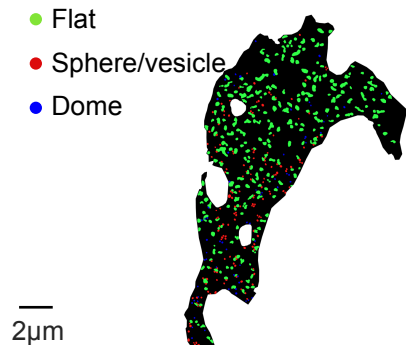

**B** Sochacki et al., 2021

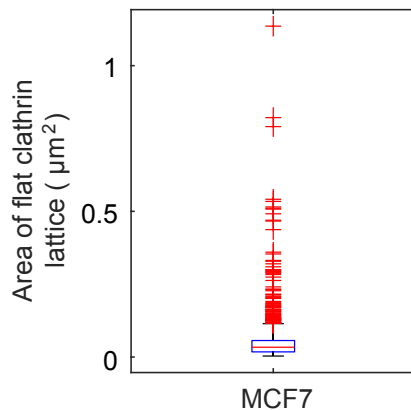

**C** Sochacki et al., 2021

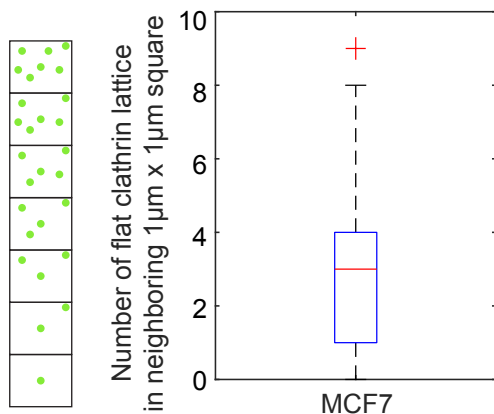

**D**

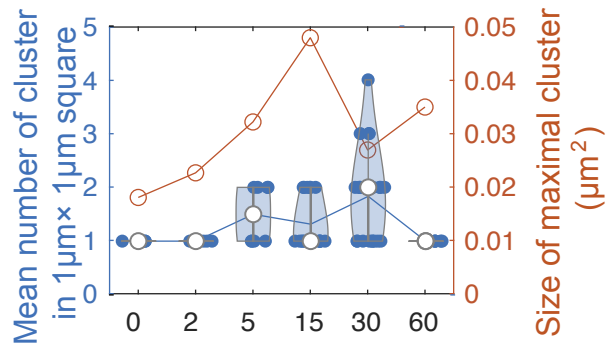

Supplement: S1 Fig — (A–C) The same plots as those in Fig 1B–1D except the cell type. (D) Same plot as in Fig 1E, except that only 1 μm × 1 μm square areas are considered instead of the entire cell membrane. Here, we focus only on the neighboring 1 μm × 1 μm square areas surrounding each cluster. (PDF) [file pcbi.1014013.s005.pdf]

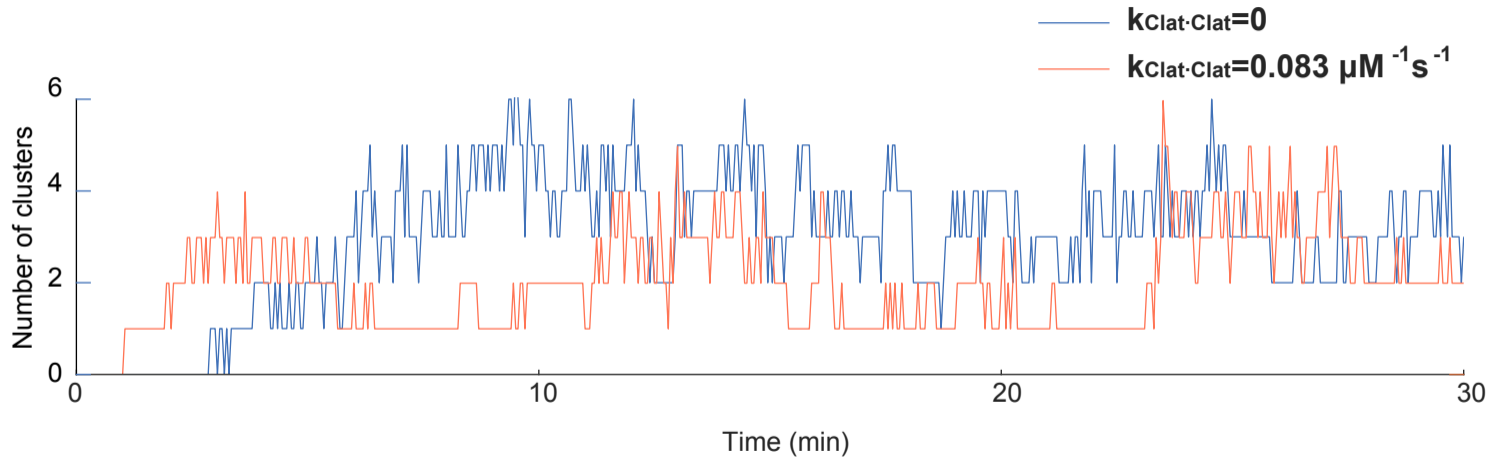

Supplement: S2 Fig — kClat−Clat represents the clathrin-clathrin binding rate when neither clathrin is bound to AP-2. Excepct the difference in kClat−Clat, the settings of model simulations are the same as that in Fig 4C. (PDF) [file pcbi.1014013.s006.pdf]

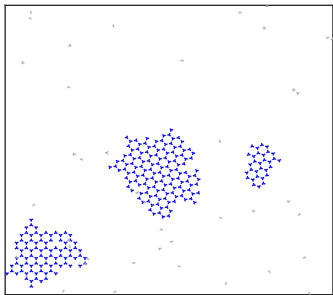

30 min

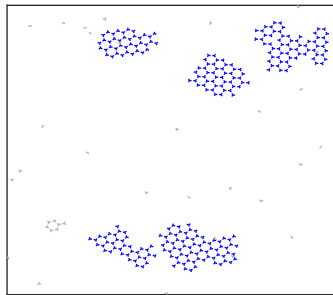

35 min

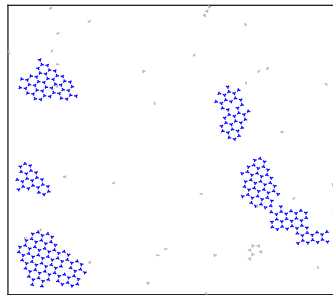

40 min

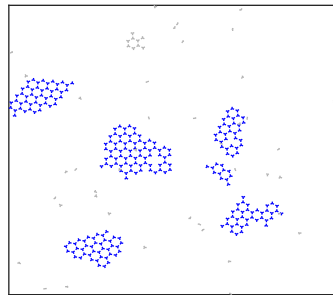

45 min

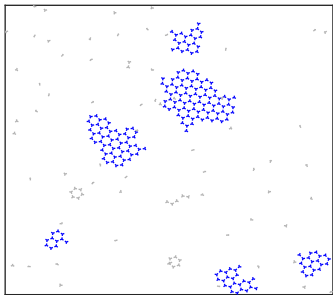

50 min

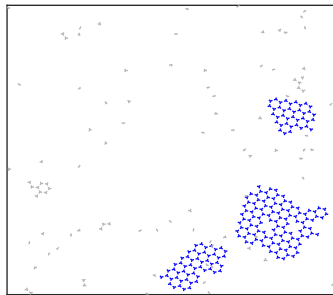

55 min

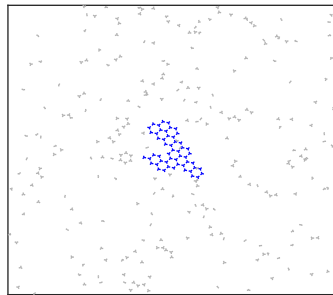

60 min

Supplement: S7 Fig — (PDF) [file pcbi.1014013.s011.pdf]

**A**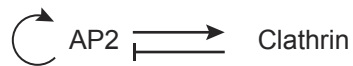**C**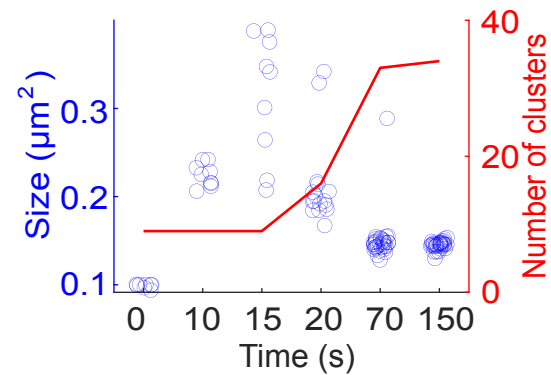**B**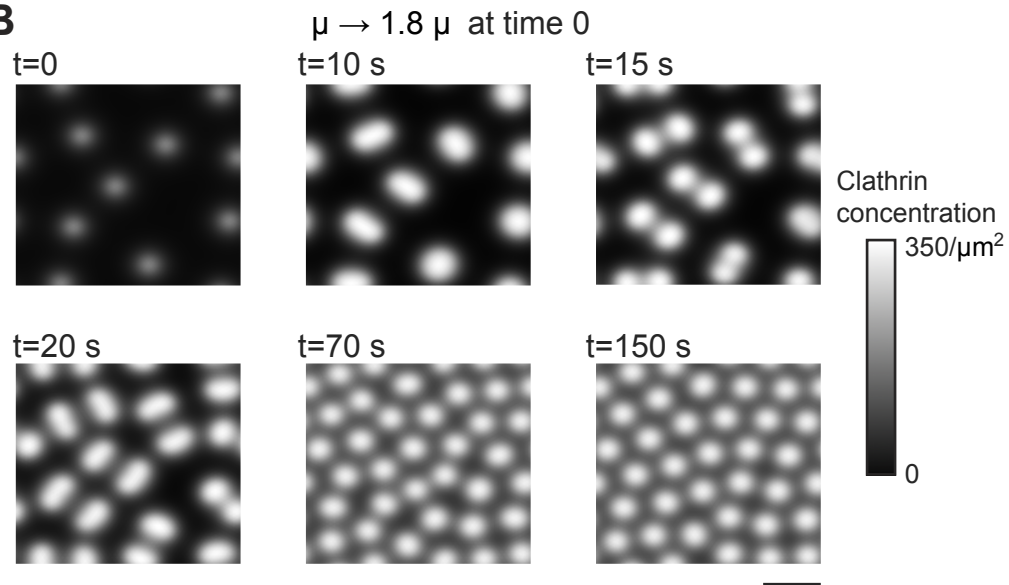**D**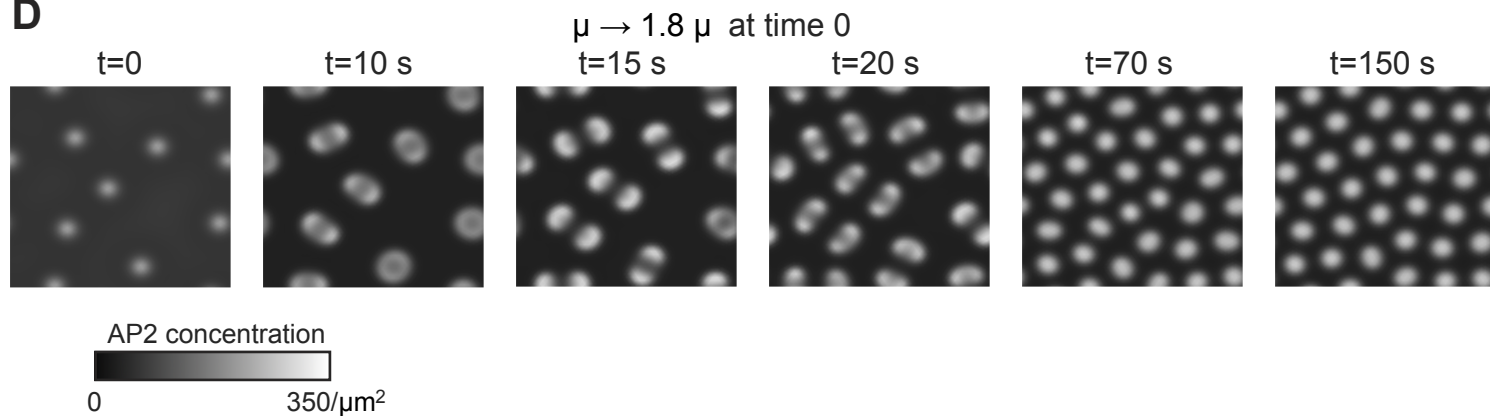

Supplement: S8 Fig — (A) Schematic of reactions between the AP-2 and the clathrin. The AP-2 improves the recruitment of itself and the clathrin to the cell membrane. Once bound to the cell membrane, the clathrin inhibits the accumulation of the AP-2 on the cell membrane due to steric repulsion. (B) The snapshots of clathrin clusters after increasing the association rate of AP-2 and membrane μ by 80% at time 0. The first plot corresponds to the Turing pattern with parameters in S3 Table. Scale bar is 1 μm. (C) The size for each clathrin cluster (left axis) and the total number of clathrin clusters (right axis) at the time points in (B). The increase in the clathrin cluster size only occurs between 0 and 15 seconds, while the increase of the total number of clathrin clusters occurs after 15 seconds. (D) The same plot as that in B, except that the AP-2 concentration is shown instead of the clathrin concentration. (PDF) [file pcbi.1014013.s012.pdf]
