# Supplementary material for: Dynamics of the formation of flat clathrin lattices in response to growth factor stimulus
Source: PLoS Comput Biol. 2026 Mar 11;22(3):e1014013. doi: 10.1371/journal.pcbi.1014013 (PMC13012621; doi:10.1371/journal.pcbi.1014013)
Supplement: S3 Fig — (A) The distribution of clathrin (blue or gray) and AP-2 (red) at 30 minutes, simulated from an initial condition where the molecules are randomly distributed. Clathrin is shown in blue or gray, depending on whether it is membrane-bound. 45 clathrin-bound AP-2 molecules are labeled in red, while the remaining 55 AP-2 molecules are not shown. (B) Same plot as (A), but with a different AP-2-clathrin binding rate. In this case, 95 clathrin-bound AP-2 molecules are shown in red, while the remaining 5 AP-2 molecules are not shown. See S1 Table for kinetic parameters. (PDF) [file pcbi.1014013.s007.pdf]

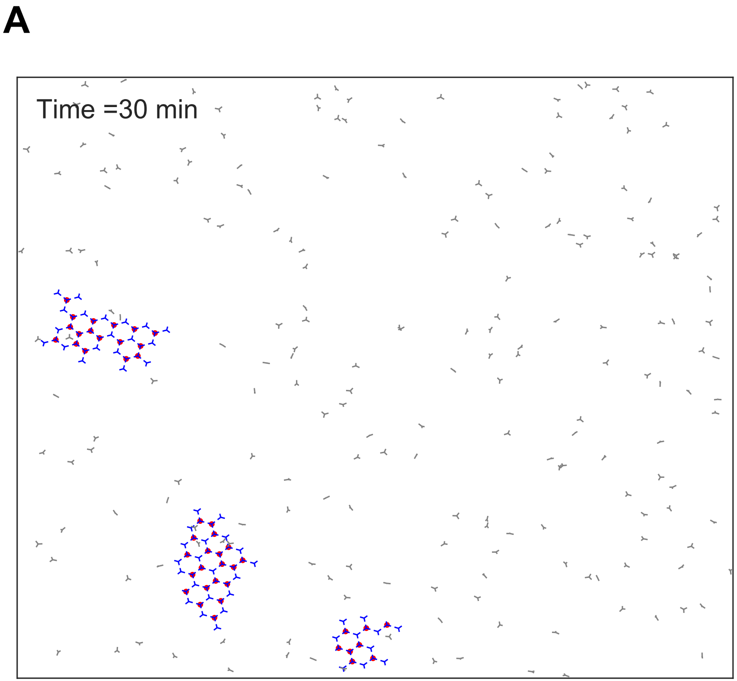

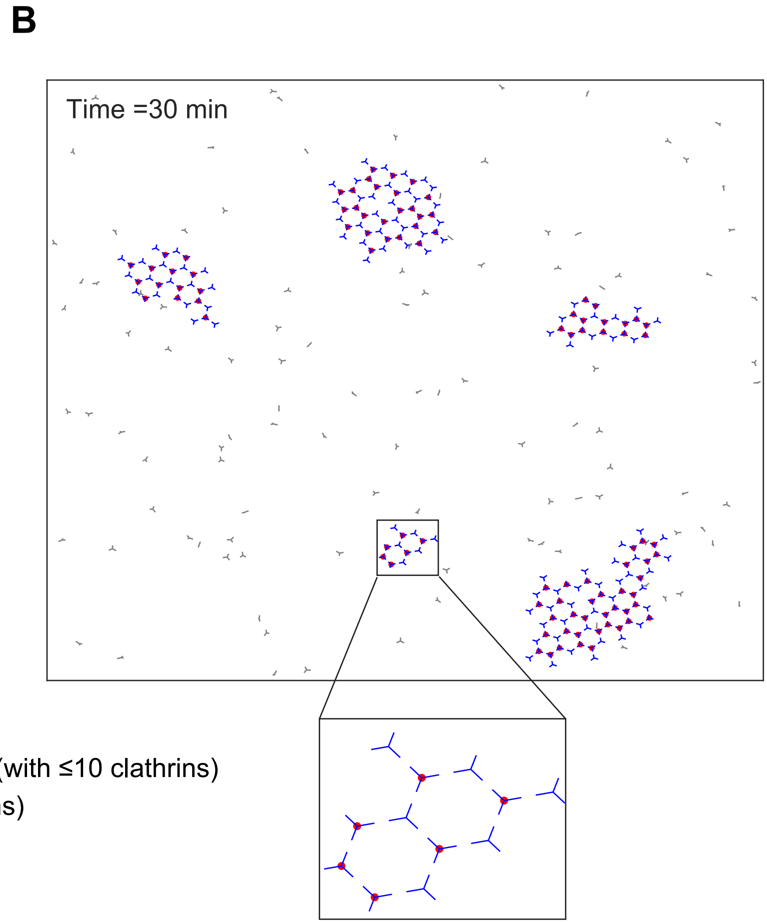

: clathrin in the cytosol or in a small membrane-bound cluster (with ≤10 clathrins)

: clathrin in a large membrane-bound cluster (with >10 clathrins)

: Clathrin-bound AP2
