# Supplementary material for: Dynamics of the formation of flat clathrin lattices in response to growth factor stimulus
Source: PLoS Comput Biol. 2026 Mar 11;22(3):e1014013. doi: 10.1371/journal.pcbi.1014013 (PMC13012621; doi:10.1371/journal.pcbi.1014013)
Supplement: S4 Fig — (A) Distributions of mean cluster size (upper panel) and dwelling time (lower panel) for all data points, obtained from the simulations shown in Fig 2. (B–E) Same plots as in (A), but restricted to data points with cluster numbers of 1 (B), 2 (C), 3 (D), and 4 (E), respectively. (F) The mean value of the cluster size in panels (B–E). S1 represents the mean cluster size in panel (B), corresponding to the highest dashed line. The dashed lines, from the second highest to the lowest, represent 12S1, 13S1, and 14S1, respectively. (G) Standard deviation of the mean cluster size in panels (A–E). (PDF) [file pcbi.1014013.s008.pdf]

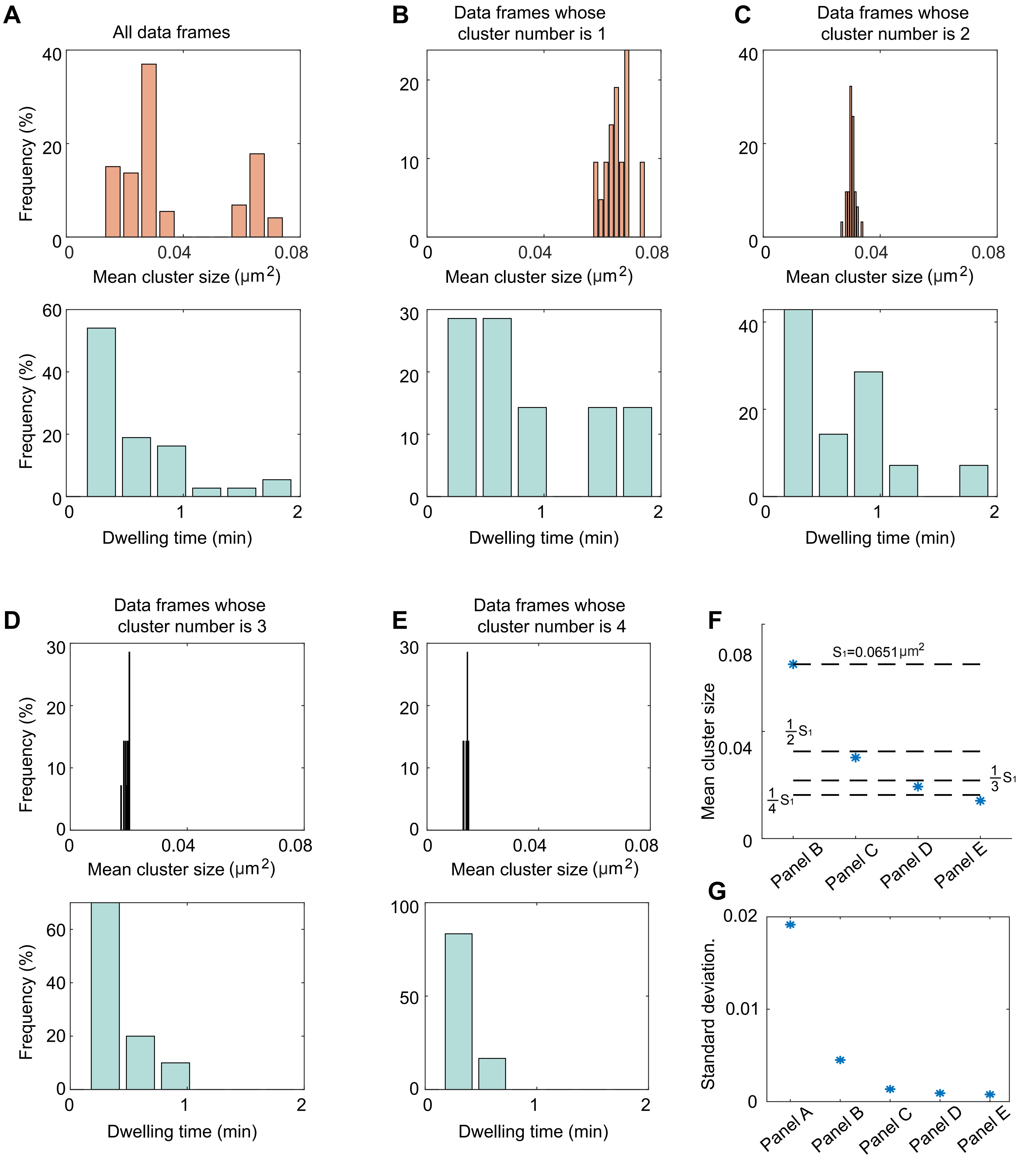
A
All data frames
Frequency (%)
Mean cluster size (μm2)
Dwelling time (min)
B
Data frames whose cluster number is 1
C
Data frames whose cluster number is 2
D
Data frames whose cluster number is 3
E
Data frames whose cluster number is 4
F
S1=0.0651μm2
1/2 S1
1/3 S1
1/4 S1
Mean cluster size
Panel B
Panel C
Panel D
Panel E
G
Standard deviation.
Panel A
