# Supplementary material for: Dynamics of the formation of flat clathrin lattices in response to growth factor stimulus
Source: PLoS Comput Biol. 2026 Mar 11;22(3):e1014013. doi: 10.1371/journal.pcbi.1014013 (PMC13012621; doi:10.1371/journal.pcbi.1014013)
Supplement: S5 Fig — (A–F) The same plots as those in Fig 3 except that a low clathrin-clathrin binding rate is used. (PDF) [file pcbi.1014013.s009.pdf]

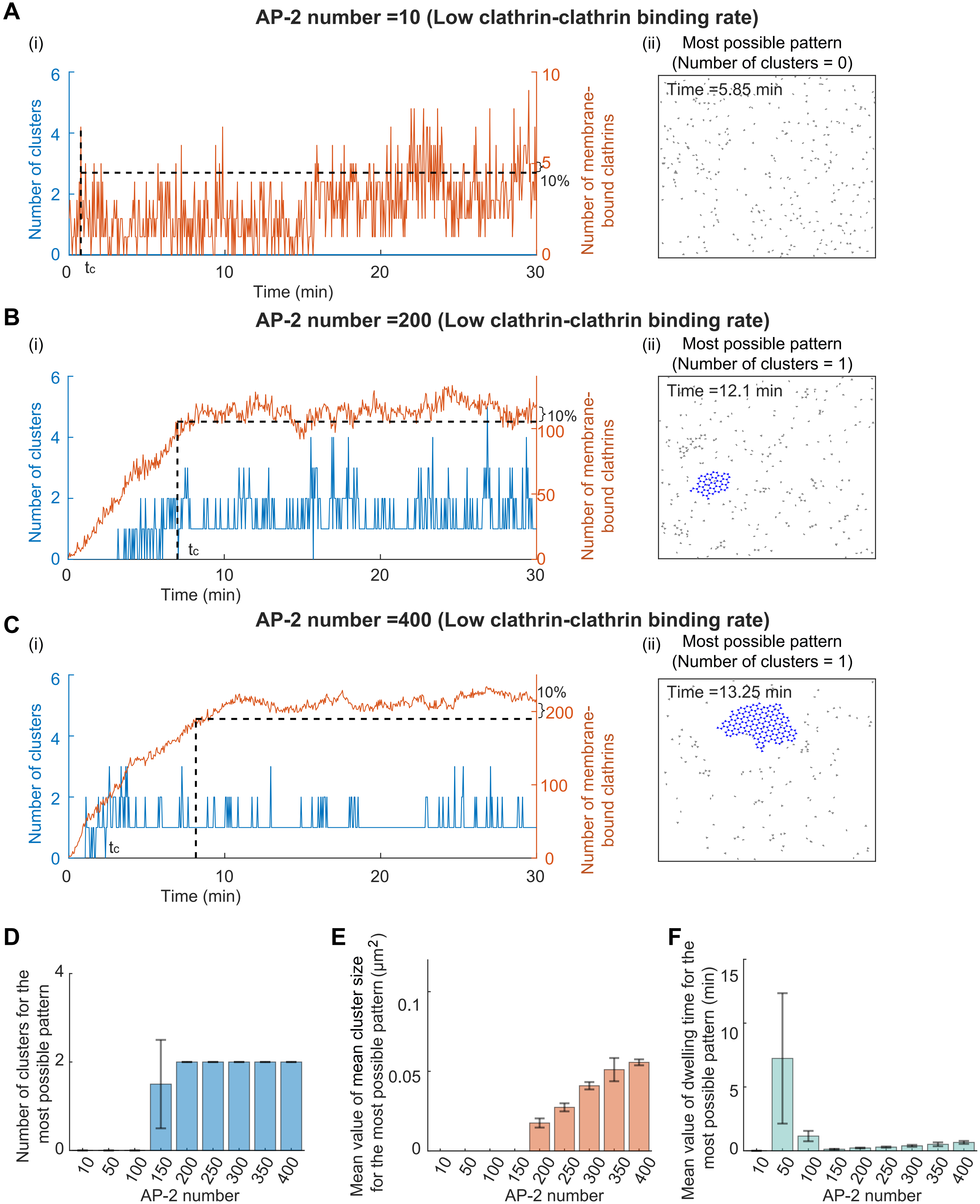

A
AP-2 number =10 (Low clathrin-clathrin binding rate)
(i)
Number of clusters
Number of membrane-bound clathrins
10%
tc
Time (min)
(ii) Most possible pattern
(Number of clusters = 0)
Time =5.85 min
B
AP-2 number =200 (Low clathrin-clathrin binding rate)
(i)
Number of clusters
Number of membrane-bound clathrins
10%
tc
Time (min)
(ii) Most possible pattern
(Number of clusters = 1)
Time =12.1 min
C
AP-2 number =400 (Low clathrin-clathrin binding rate)
(i)
Number of clusters
Number of membrane-bound clathrins
10%
tc
Time (min)
(ii) Most possible pattern
(Number of clusters = 1)
Time =13.25 min
D
Number of clusters for the most possible pattern
AP-2 number
E
Mean value of mean cluster size for the most possible pattern (μm²)
AP-2 number
F
Mean value of dwelling time for the most possible pattern (min)
AP-2 number
