# Supplementary material for: Dynamics of the formation of flat clathrin lattices in response to growth factor stimulus
Source: PLoS Comput Biol. 2026 Mar 11;22(3):e1014013. doi: 10.1371/journal.pcbi.1014013 (PMC13012621; doi:10.1371/journal.pcbi.1014013)
Supplement: S6 Fig — (A–B) Simulated clathrin dynamics when the AP2-clathrin binding rate kAP-2·Clat is 0.5 × 0.0012 μM−1s−1 (A), 10 × 0.0012 μM−1s−1 (B) Parameters except kAP-2·Clat are fixed. The initial condition is that clathrins are randomly distributed in the simulation domain. In the left panel, the number of clusters (in blue) and the number of membrane-bound clathrins (in red) are shown as a function of time. Panels on the right showed the most possible pattern. (C) The cluster number for the most possible pattern when increasing kAP−2·Clat. Data were shown as mean±SD, where SD means standard deviation. (PDF) [file pcbi.1014013.s010.pdf]

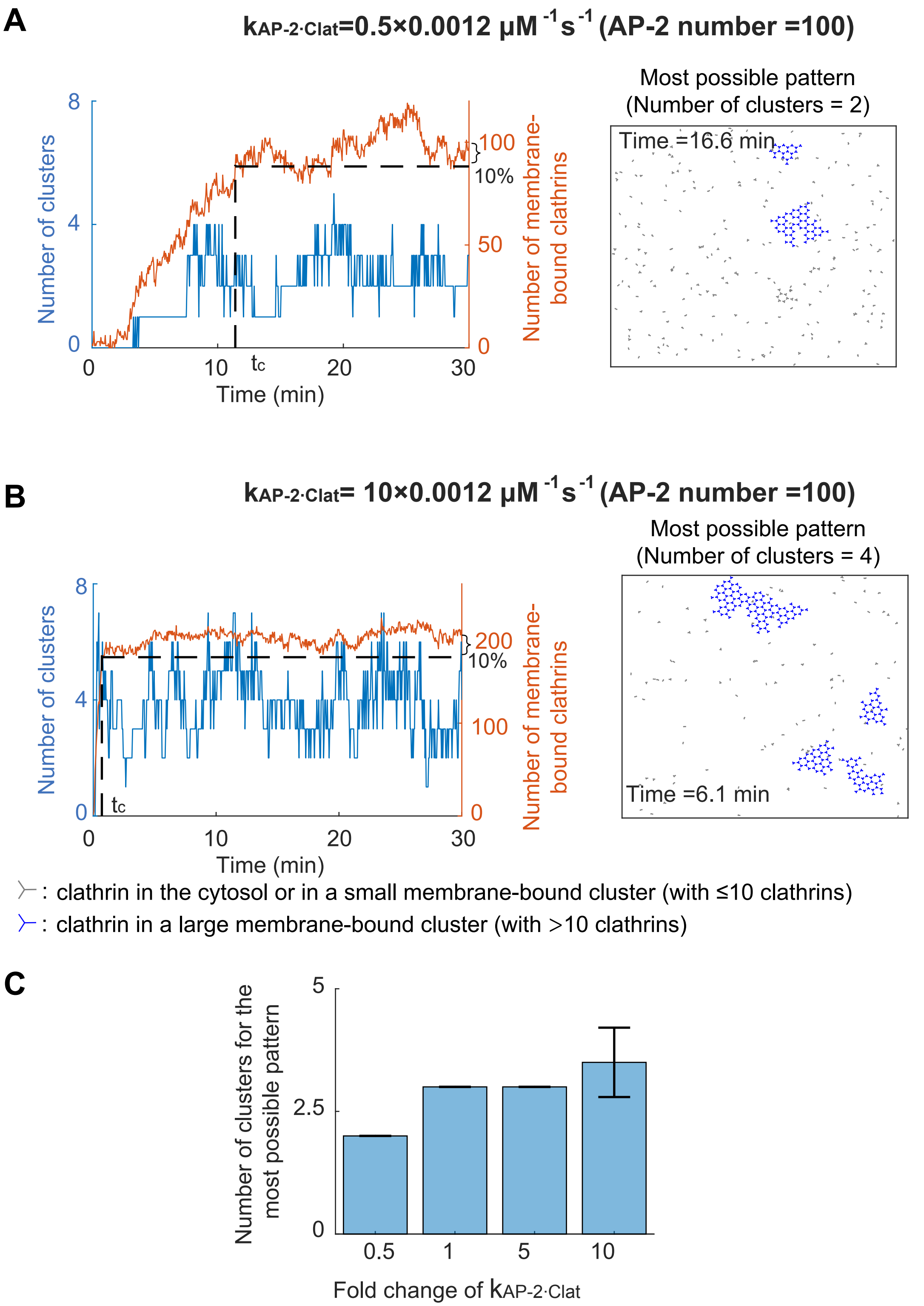
A
kAP-2·Clat=0.5×0.0012 μM -1s -1 (AP-2 number =100)
Number of clusters
Number of membrane-bound clathrins
Time (min)
tc
10%
Most possible pattern
(Number of clusters = 2)
Time =16.6 min
B
kAP-2·Clat= 10×0.0012 μM -1s -1 (AP-2 number =100)
Number of clusters
Number of membrane-bound clathrins
Time (min)
tc
10%
Most possible pattern
(Number of clusters = 4)
Time =6.1 min
: clathrin in the cytosol or in a small membrane-bound cluster (with ≤10 clathrins)
: clathrin in a large membrane-bound cluster (with >10 clathrins)
C
Number of clusters for the most possible pattern
Fold change of kAP-2·Clat
0.5
1
5
10
