# Supplementary material for: Dynamics of the formation of flat clathrin lattices in response to growth factor stimulus
Source: PLoS Comput Biol. 2026 Mar 11;22(3):e1014013. doi: 10.1371/journal.pcbi.1014013 (PMC13012621; doi:10.1371/journal.pcbi.1014013)
Supplement: S9 Fig — (A–C) Same plots as Fig 3A–3C, except with three clathrin-AP-2 binding sites instead of one. Here, only the most possible pattern was shown, while other patterns were not shown. (D–F) Same plots as Fig 3D–3F, but with the results for one and three clathrin-AP-2 binding sites plotted. (PDF) [file pcbi.1014013.s013.pdf]

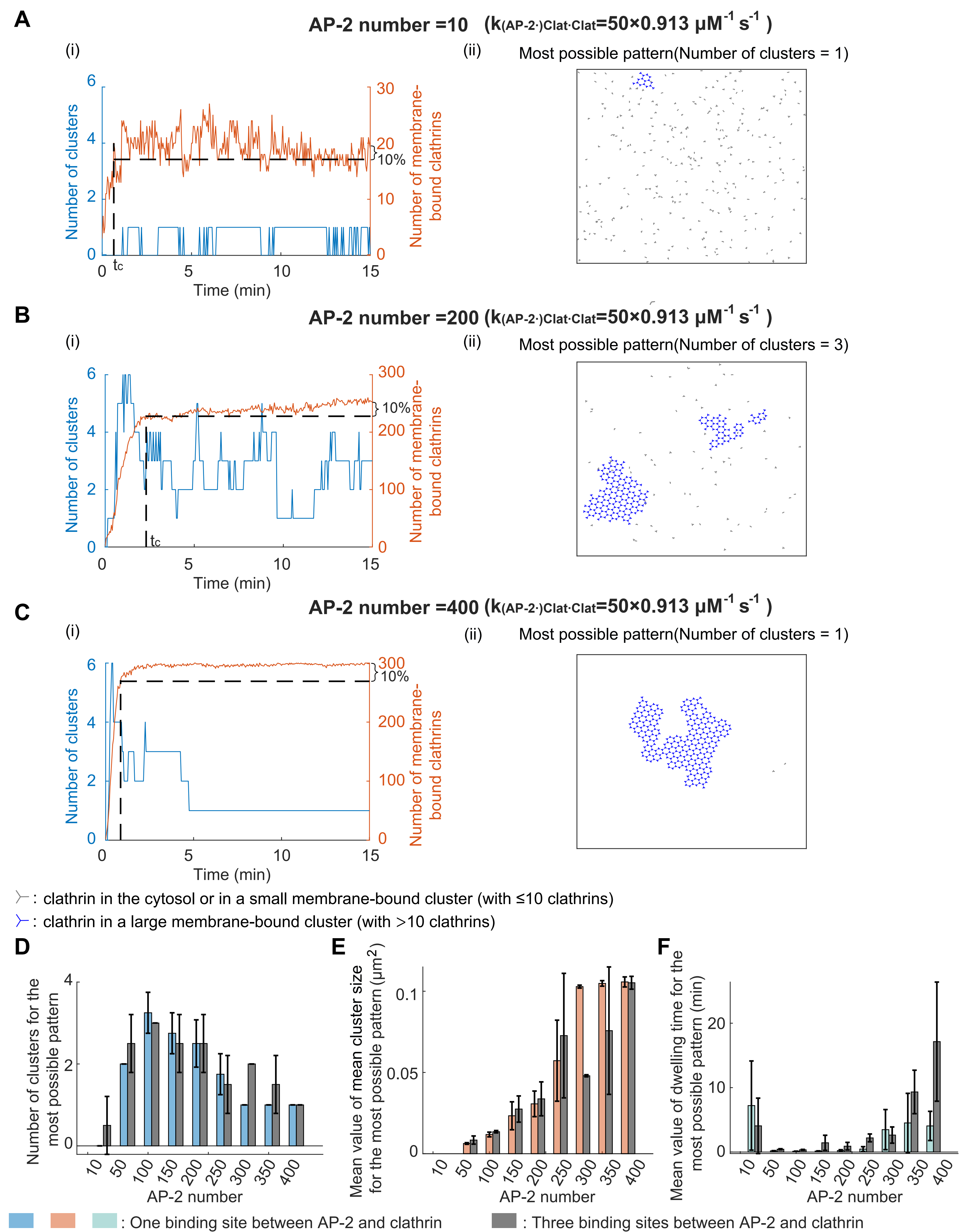

A
AP-2 number =10 (k(AP-2·)Clat·Clat=50×0.913 μM-1 s-1)
(i)
(ii) Most possible pattern(Number of clusters = 1)
Number of clusters
Number of membrane-bound clathrins
10%
tc
Time (min)
B
AP-2 number =200 (k(AP-2·)Clat·Clat=50×0.913 μM-1 s-1)
(i)
(ii) Most possible pattern(Number of clusters = 3)
Number of clusters
Number of membrane-bound clathrins
10%
tc
Time (min)
C
AP-2 number =400 (k(AP-2·)Clat·Clat=50×0.913 μM-1 s-1)
(i)
(ii) Most possible pattern(Number of clusters = 1)
Number of clusters
Number of membrane-bound clathrins
10%
Time (min)
: clathrin in the cytosol or in a small membrane-bound cluster (with ≤10 clathrins)
: clathrin in a large membrane-bound cluster (with >10 clathrins)
D
Number of clusters for the most possible pattern
AP-2 number
E
Mean value of mean cluster size for the most possible pattern (μm2)
AP-2 number
F
Mean value of dwelling time for the most possible pattern (min)
AP-2 number
: One binding site between AP-2 and clathrin
: Three binding sites between AP-2 and clathrin
