# Supplementary material for: Dynamics of the formation of flat clathrin lattices in response to growth factor stimulus
Source: PLoS Comput Biol. 2026 Mar 11;22(3):e1014013. doi: 10.1371/journal.pcbi.1014013 (PMC13012621; doi:10.1371/journal.pcbi.1014013)
Supplement: S10 Fig — (A–C) Same plots as Fig 4A–4C, except with three clathrin-AP-2 binding sites instead of one. Here, only the most possible pattern was shown, while other patterns were not shown. (D–E) Same plots as Fig 4D–4E, but with the results for one and three clathrin-AP-2 binding sites plotted. (F) Example of the locations of clathrin (blue) and AP-2 (red). (PDF) [file pcbi.1014013.s014.pdf]

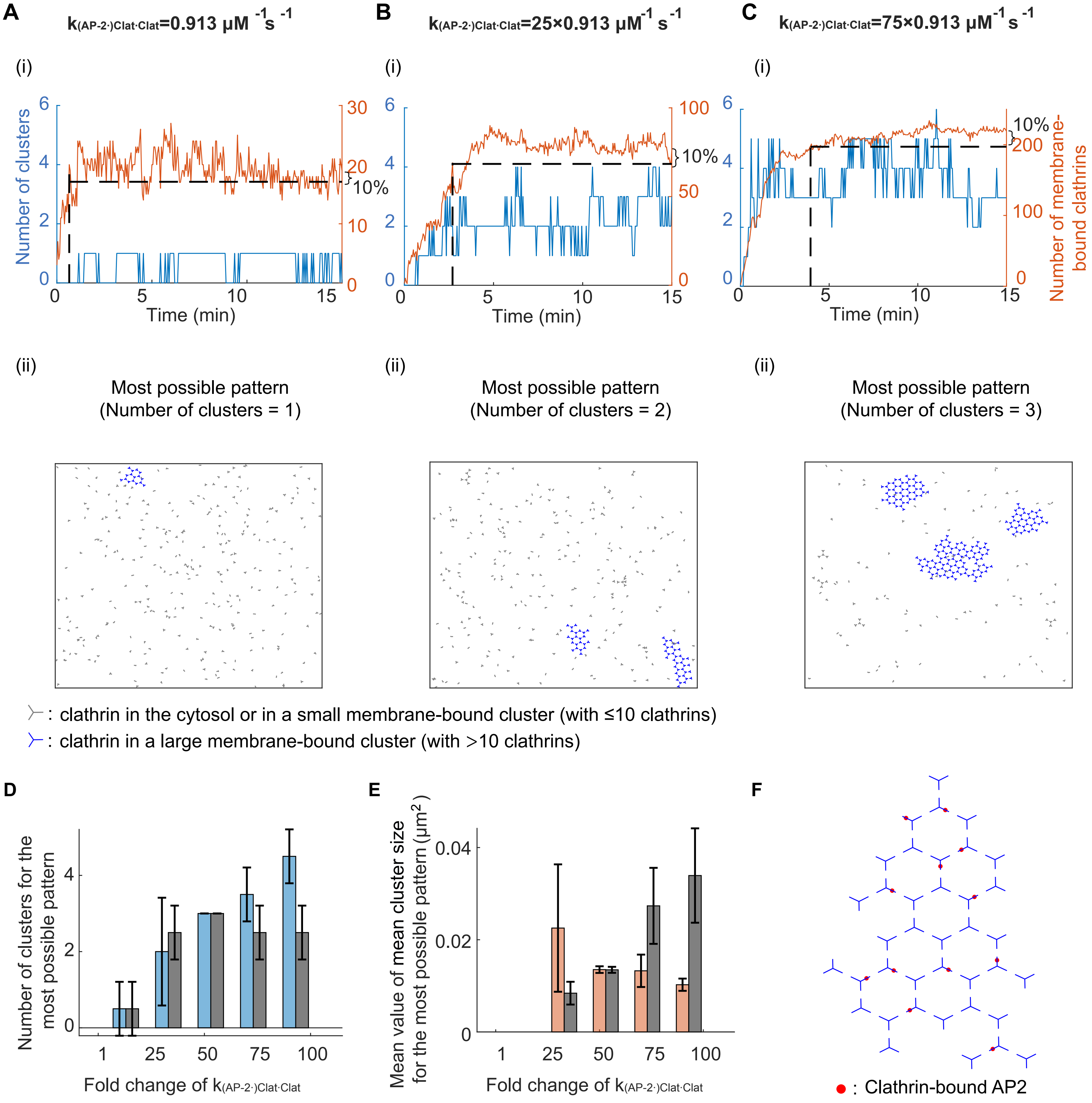

A
$k_{(AP\text{-}2\cdot)Clat\cdot Clat}$=0.913 μM^{-1}s^{-1}
B
$k_{(AP\text{-}2\cdot)Clat\cdot Clat}$=25×0.913 μM^{-1} s^{-1}
C
$k_{(AP\text{-}2\cdot)Clat\cdot Clat}$=75×0.913 μM^{-1} s^{-1}
(i)
Number of clusters
Number of membrane-bound clathrins
Time (min)
10%
(ii)
Most possible pattern
(Number of clusters = 1)
Most possible pattern
(Number of clusters = 2)
Most possible pattern
(Number of clusters = 3)
: clathrin in the cytosol or in a small membrane-bound cluster (with ≤10 clathrins)
: clathrin in a large membrane-bound cluster (with >10 clathrins)
D
Number of clusters for the most possible pattern
Fold change of $k_{(AP\text{-}2\cdot)Clat\cdot Clat}$
E
Mean value of mean cluster size for the most possible pattern (μm^2)
Fold change of $k_{(AP\text{-}2\cdot)Clat\cdot Clat}$
F
: Clathrin-bound AP2
