# Supplementary material for: Dynamics of the formation of flat clathrin lattices in response to growth factor stimulus
Source: PLoS Comput Biol. 2026 Mar 11;22(3):e1014013. doi: 10.1371/journal.pcbi.1014013 (PMC13012621; doi:10.1371/journal.pcbi.1014013)
Supplement: Attachment — Submitted filename: Response_letter.pdf [file pcbi.1014013.s016.pdf]

## Reviewer #1 (Comments to the Author):

Multiple flat clathrin lattices exist on the cell membrane serving as signaling hubs after epidermal growth factor stimulation. This paper used a particular-based model to simulate the assembly and disassembly of flat clathrin lattices to capture these dynamics. The results are quite interesting and could be useful to reveal the mechanisms underlying the formation of flat clathrin lattices and how epidermal growth factor regulate their dynamics. I suggest the authors to consider the following comments and incorporate the corresponding revisions before the manuscript can be accepted for publication.

### Comments

*1. In the introduction section, the authors should also mention a little bit background about the application of particle-based simulations to other membrane protein systems, such as TNF-induced receptor clustering by Su et al.*

**Response:** We thank the reviewer for this valuable suggestion. We agree that particle-based simulations have broad applications to membrane protein systems beyond clathrin.

**Actions taken:** In the revision, we have added a paragraph in the Introduction section (lines 65-75) to introduce the application of particle-based simulations to other cell membrane protein assembles, including TNF receptor clustering, HIV-1 Gag lattice assembly, and Cdc42 assembly.

*2. In the model development part, there are several issues needed to be clarified. First, whether periodic boundary conditions were applied along x and y directions? How about the direction that is perpendicular to the cell membrane?*

**Response:** We apologize for the lack of clarity. In the model, the boundaries are six surfaces surrounding the simulation domain (two along each of the x, y, and z axes). All these boundaries are set as reflecting boundaries, so the molecule will be reflected back into the volume if it hits the boundary. Specifically, if a molecule's tentative position at the next step exceeds a boundary due to chemical reactions or diffusion, for example, the right boundary at $x = L$, the $y$- and $z$-coordinates are updated as usual, while the $x$-coordinate is reflected and set to $2L - pos_1^{new}$, where $pos_1^{new}$ is the $x$-coordinate of the tentative position. However, an association event is rejected if it would cause the clathrin cluster to extend beyond the simulation domain.

**Actions taken:** In the revision, to clarify the use of reflecting boundaries, we added a paragraph in the Model Development section (lines 207-215). In this paragraph, we describe the implementation of reflecting boundaries.

*3. How to describe the 2D diffusion of AP-2 in the cell membrane? How to determine its 2D diffusion coefficient in the living cell?*

**Response:** In the original manuscript, to constrain AP-2 on the cell membrane, its translational diffusion coefficient along the $z$ axis ($D_{AP\text{-}2,z}$) was set to zero, and so were its rotational diffusion coefficients around the $x$ and $y$ axes ($D_{R,AP\text{-}2,x}$ and $D_{R,AP\text{-}2,y}$, respectively). These settings ensure that AP-2 is always on the cell membrane. Once clathrins bind to AP-2, the cluster diffusion coefficients are given by applying $D_{\text{Cluster}} = \left[\sum_{i\in\text{cluster}} D_i^{-1}\right]^{-1}$ and $D_{R,\text{Cluster}} = \left[\sum_{i\in\text{cluster}} D_{R,i}^{-\frac{1}{3}}\right]^{-3}$ to directions $x$,$y$, and $z$ (see the revised manuscript). According to these equations, the cluster has zero translational diffusion along the $z$ axis and zero rotational diffusion around the $x$ and $y$ axes, ensuring that the cluster always stays on the cell membrane.

Furthermore, in cells, diffusion coefficients are usually measured by labeling particles of interest with fluorescent proteins, monitoring their fluorescence (e.g., via fluorescence recovery after photobleaching), and extracting diffusion coefficients from the experimental data using mathematical models [1]. For particles that are spherical and diffuse freely, the Stokes–Einstein equation can provide a good estimate of the diffusion coefficient. However, for membrane-associated particles whose diffusion is restricted to the cell membrane, other mathematical models are used [2, 3, 4]. Meanwhile, fluorescence-labeling strategies, microscopy techniques, and mathematical models have been continuously developed to improve the accuracy of estimating diffusion coefficient measurements for various particles [5, 6, 7].

**Actions taken:** In the revision, we have added two paragraphs (lines 132-169) in the Model Development section to describe how diffusion coefficients are determined for individual AP-2, clathrin molecules, and assembled clusters. In addition, we briefly mentioned how diffusion coefficients have been measured in other systems, emphasizing that different systems may require different relationships to extract the diffusion coefficient.

*4. The simulations can directly give the cluster number, cluster size and dwelling time as measurable quantities. If any of these are comparable to the available experimental data?*

**Response:** Thank you for the good question. Experiments in [8] showed that, in the absence of stimulation, cluster sizes ranged from $0.01\mu m^2$ to $0.3\mu m^2$ in HeLa and U87 cells, and from $0.01\mu m^2$ to $1.3\mu m^2$ in MCF7 cells. Despite these range differences, the mean cluster size was approximately $0.05\mu m^2$ in all three types of cells. In our simulations, the cluster sizes ranged from $0.001\mu m^2$ to $0.1\mu m^2$, which is around the mean cluster size observed in the experimental data.

For the cluster number, data in [8] showed the cluster number in $1\mu m \times 1\mu m$ square ranges from 0 to 10 in HeLa, U87, and MCF7 cells, with 1 or 2 clusters occurring most frequently. In our simulations, the bottom of the simulation domain, representing the cell membrane, also is a $1\mu m \times 1\mu m$ square. Within this region, the simulated clathrins formed 0 to 12 clusters, which is in agreement with the cluster numbers observed experimentally.

As for dwelling time, Grove et al. reported that flat clathrin lattices are long-lived, typically lasting more than 10 minutes [9]. However, these measurements were based on the intensity of fluorescence-labeled clathrin, which cannot distinguish individual flat clathrin lattices due to low resolution. In contrast, the electron microscopy technique used in [10] provides sufficient resolution to distinguish individual flat clathrin lattices, but it cannot be applied to monitor the dynamics of the cluster, as the cells must be fixed during imaging. Therefore, large flat clathrin lattices may have long dwelling times more than 10 minutes, but it remains unclear whether small lattices exhibit short or long dwelling times. In our simulations, the dwelling time ranges from 0.05 to 10 minutes depending on cluster size.

**Actions taken:** In the revision, we have added a paragraph (lines 394-411) in the Discussion section to briefly describe the cluster number, cluster size, and dwelling time observed experimentally and obtained from our simulations. In this paragraph, we summarize that the cluster number and size from simulations are consistent with experimental observations. Furthermore, we note that current techniques may be insufficient to accurately measure the dwelling time of individual flat clathrin lattices.

*5. In the results part 3.3, the authors adjusted the binding rate between clathrins. Is this the association rate or dissociation rate?*

**Response:** Sorry for the confusion. Throughout the manuscript, "binding rate" refers to the

association rate, whereas the term "unbinding rate" refers to the dissociation rate.

**Actions taken:** In the manuscript, to clarify that the binding rate corresponds to the association rate, we added the following sentence in Results Section 3.3 (lines 297-298): Throughout this work, the binding and unbinding rates refer to the association and dissociation rates, respectively.

*6. It would be interesting to see how the dynamics of assembly and disassembly would be regulated by changing the binding rate between clathrin and AP-2.*

**Response:** We thank the reviewer for this suggestion.

**Actions taken:** To illustrate how the binding rate between AP-2 and clathrin affects clathrin dynamics, in the revision, we have added a new Figure S6. In this figure, we varied the AP-2-clathrin binding rate while keeping the other kinetic parameters fixed and found that an increase in the AP-2-clathrin binding rate leads to an increase in the number of clusters. Therefore, the role of the AP-2-clathrin binding rate is similar to that of the clathrin-clathrin binding rate: a higher binding rate leads to an increase in the number of clusters.

*7. In 3.5, the authors investigated the trend of clathrin lattices under EGF stimulus. One concern resulted from the fact that only four scenarios were tested and the authors argued that the last combination can lead to the experimentally observed trend. This, however, cannot exclude other possible conditions which might also lead to the similar behavior of clathrin lattice assembly.*

**Response:** We agree with the reviewer that the four scenarios we tested in the manuscript cannot exclude other possible conditions. We appreciate the reviewer's insight and recognize this limitation.

**Actions taken:** To acknowledge this limitation, in the revision, we added the following text in the Discussion section (lines 439-443): However, it should be noted that other possible conditions may also generate the experimentally observed EGF-triggered cluster dynamics, such as changes in the clathrin-clathrin unbinding rate or the binding rate between AP-2 and clathrin, which were not tested in this study. Future detailed simulations will be necessary to explore all the potential mechanisms underlying the EGF-triggered FCL changes.

## Reviewer #2 (Comments to the Author):

The authors present a simulation study on the number and average size of flat clathrin lattices, exploring the effects of three parameters in particular. These three parameters are then used to explain the experimentally observed growth of lattices, and their subsequent return to the equilibrium state, when the cell is stimulated with epidermal growth factor. The study shows some interesting trends, but also raises a number of questions.

*1. The authors reduce the number of clathrin-AP2 binding sites to one, which "we assume that it does not quantitatively affect the results" (p5). But, since this reduction rules out cooperative effects of clathrin binding to multiple AP2, it may even affect the results qualitatively.*

**Response:** Thank you for the good question. In response to this question, we conducted new simulations where there are three binding sites between clathrin and AP-2. In this system, we tested the effect of AP-2 (new Figure S9) and clathrin-clathrin binding rate (new Figure S10) on the clathrin dynamics. The new results show that, although three clathrin-AP-2 binding sites affect the cluster properties quantitatively, some properties are maintained qualitatively. For example, the effects of AP-2 number on cluster number, cluster size, and dwelling time, as well as the impact of clathrin-clathrin binding rate on cluster number, remain similar.

**Actions taken:** To show the three clathrin-AP-2 binding sites not qualitatively affect the result, we conducted new simulation with three clathrin-AP-2 binding sites, with the results shown in new Figures S9 and S10. We also added the following text in the Discussion section "To ensure that the reducing the clathrin-AP-2 binding sites not quantitatively affect the result, we tested effect of AP-2 number (Figure S9) and clathrin-clathrin binding sites (Figure S10) when there are three clathrin-AP-2 binding sites. Though the exact number of clusters, cluster size, and dwelling time are different between the systems with one and three clathrin-AP-2 binding sites, increasing the number of AP-2 exhibits a similar trend (Figure S9D-F): the transition from no clusters to short-lived, multiple small clusters, and then to a long-lasting, single giant cluster. In addition, in the system with three clathrin-AP-2 binding sites, increasing the clathrin-clathrin binding rate leads to an increase in the number of clusters (Figure S10D-F), which is consistent with the result observed when there is only one clathrin-AP-2 binding site. Therefore, we assume that the simplification from three clathrin-AP-2 binding sites to one only qualitatively affects the results."

*2. The peculiar addition rule for diffusion coefficients, page 5, has its consequences. The translation diffusion coefficient of a spherical cluster scales inversely proportional with its radius (Stokes-Einstein), not with the number of monomers. Its rotation diffusion diffusion coefficient scales inversely proportional with the radius cubed (Stokes-Einstein-Debye), not with the number of monomers cubed. Consequently, the simulated clusters diffuse too slowly (which following section 3.4 affects flat lattice formation).*

**Response:** We apologize for the confusion. We agree that the translation diffusion coefficient of a spherical cluster scales inversely proportional with its radius, while its rotation diffusion coefficient scales inversely proportional with the radius cubed. However, by combining these facts with the assumption that the cluster radius is the sum of the radii of its components, we can obtain $D_{\text{Cluster}} = \left[\sum_{i\in\text{cluster}} D_i^{-1}\right]^{-1}$, $D_{R,\text{Cluster}} = \left[\sum_{i\in\text{cluster}} D_{R,i}^{-\frac{1}{3}}\right]^{-3}$ for the translational and rotational diffusion coefficients (also used in [11]), respectively. Next, we show the detailed derivations for diffusion in one direction (the manuscript considers $x$, $y$, and $z$).

First, according to the Stokes-Einstein equations, once the translational diffusion coefficient $D$ and rotational diffusion coefficient $D_R$ are given, the effective hydrodynamic radii for translation and rotation can be expressed as

$$r = \frac{k_B T}{6\pi\eta} D^{-1}, \qquad r_R = \frac{k_B T}{8\pi\eta} D_R^{-\frac{1}{3}},$$

respectively. $k_B$ is the Boltzmann constant, $T$ is the absolute temperature, and $\eta$ is the medium viscosity. We assumed that the effective radii for translation and rotation can be different. Then, assuming that the cluster radius is the sum of the radii of its components, i.e.,

$$r_{cluster} = \sum_{i \in \text{cluster}} r_i, r_{R,cluster} = \sum_{i \in \text{cluster}} r_{R,i},$$

Thus, the cluster translational diffusion coefficient is given by

$$D_{\text{Cluster}} = \frac{k_B T}{6\pi\eta r_{\text{cluster}}} = \frac{k_B T}{6\pi\eta \sum_{i \in \text{cluster}} \frac{k_B T}{6\pi\eta} D_i^{-1}} = \left[ \sum_{i \in \text{cluster}} D_i^{-1} \right]^{-1}.$$

Similarly, the cluster rotational diffusion coefficient is given by

$$D_{R,\text{Cluster}} = \frac{k_B T}{8\pi\eta r^3_{\text{cluster}}} = \frac{k_B T}{8\pi\eta \sum_{i \in \text{cluster}} \frac{k_B T}{8\pi\eta} D_{R,i}^{-\frac{1}{3}}} = \left[ \sum_{i \in \text{ cluster}} D_{R,i}^{-\frac{1}{3}} \right]^{-3}.$$

In summary, the equations for cluster diffusion coefficients used in the manuscript result from the Stokes–Einstein equations combined with the assumption that the cluster radius are the sum of the radii of all components (also see the point 3 from the reviewer 1).

**Actions taken:** To illustrate the derivation of $D_{\text{Cluster}} = \left[ \sum_{i \in \text{cluster}} D_i^{-1} \right]^{-1}$, $D_{R,\text{Cluster}} = \left[ \sum_{i \in \text{cluster}} D_{R,i}^{-\frac{1}{3}} \right]^{-3}$, in the revision, we added two new paragraphs (lines 132-169) in the Model development section.

*3. Why are average cluster size and dwelling time calculated only for systems containing the most probable number of clusters? Why introduce this bias, in stead of analyzing all frames (after the equilibration period $t_c$)?*

**Response:** We thank the reviewer for this insightful question. In the revision, we performed the new analyzes as suggested (new Figure S4). Specifically, we calculated the average cluster size and dwelling time not only for all data frames (new Figure S4A) but also for subsets of data frames with the same cluster number (new Figure S4B–E). Note that in Figure 2 in the original manuscript, the most possible pattern corresponds to the subset of data frames with cluster number 2.

We found that when the cluster number is 1, 2, 3, and 4, the average cluster sizes are approximately $S_1$, $\frac{1}{2}S_1$, $\frac{1}{3}S_1$, and $\frac{1}{4}S_1$, respectively (new Figure S4F). Here, $S_1$ represents the mean cluster size for data frames whose cluster number is 1. Thus, it seems that after $t_c$, the number of membrane-bound clathrins remains relatively constant, but clathrins undergo dynamic disassembly and reassembly, reorganizing into sub-clusters of similar size. Consequently, compared with data

frames with the same cluster number, all data frames show a larger standard deviation of the average cluster size, since the cluster size can be $S_1$, $\frac{1}{2}S_1$, $\frac{1}{3}S_1$, and $\frac{1}{4}S_1$ (new Figure S4G). Therefore, to minimize the variations in the cluster size, we only focused on the representative pattern, that is, the most pattern pattern that is likely to be observed in experiments due to its high frequency of occurrence.

**Actions taken:** In the revision, we have added a new Figure S4 to show the distributions of cluster size and dwelling time for all frames and other possible patterns. To explain why we only focused on the most possible pattern, we also added text on lines 252-261 in the first Results section. In the revised text, we explained that the main reason for not using all data frames is the large variation in the mean cluster size across all data frames.

*4. "we used a higher clathrin-clathrin binding rate than that in [30]" (p7). The notation "20 x 1.83" in table S1 suggests a 20 fold higher binding rate, but the value listed in table 1 of ref 30 is 0.083. Please specify the actual multiplication factor and a justification thereof.*

**Response:** Sorry for the confusion. Both in the manuscript and in Ref. 30, two types of clathrin–clathrin binding rates are considered: (1) when neither clathrin is bound to AP-2, and (2) when at least one clathrin is bound to AP-2. The value 0.083 in Table 1 of Ref. 30 corresponds to the rate for the first case, and the notation '20 × 1.83' in Table S1 represents the rate for the second case. A detailed explanation is provided below.

For the first case, Guo et al. (ref 30) used a rate of 0.083 $\mu M^{-1}s^{-1}$, but we used 0. This is because a lower rate results in more clusters (see the new Figure S2), which better matches the experimental data.

For the second case where at least one clathrin is bound to AP-2, Guo et al. (ref. 30) used a rate of 0.913 $\mu M^{-1}s^{-1}$ (calculated as $\exp\left(-\frac{\Delta G_{\mathrm{coop}}}{k_B T}\right) \times 0.083\ \mu M^{-1}s^{-1}$, with $\Delta G_{\mathrm{coop}} = -2.4 k_B T$), and we adopted the same value as the default rate in our manuscript. In addition, following ref. 30 (also see the text below from Page 12 in `https://github.com/mjohn218/NERDSS/blob/master/NERDSS_USER_GUIDE.pdf`), this rate was multiplied by 2 to correct for the artificial effect introduced by labeling clathrin sites. Thus, the default binding rate of two differently labeled clathrin binding sites is 1.83 $\mu M^{-1}s^{-1}$, which corresponds to the latter parameter of "20 × 1.83" mentioned by the reviewer. Nevertheless, the actual clathrin–clathrin binding rate when at least one clathrin is bound to AP-2, is 0.913 $\mu M^{-1}s^{-1}$ (half of the 1.83 $\mu M^{-1}s^{-1}$). We apologize for mistakenly labeling 0.913 $\mu M^{-1}s^{-1}$ as 1.83 $\mu M^{-1}s^{-1}$, and this has been corrected in the revision.

> binds to itself using two distinct interfaces (A(barbed end)+A(pointed end)->). However, for models such as clathrin, the distinction between an 'a' site and a 'b' site is purely a label to distinguish their coordinates in space. In reality, they are all structurally/chemically identical sites, and all interactions are possible (A(a)+A(a)->, A(a)+A(b)->, A(b)+A(b)->). To correct for this distinction arising purely due to labeling, for these specific cases, **users should input rates multiplied by two**, relative to the case where both labels are the same. For example:
>
> - A(a)+A(a) <-> A(a!1).A(a!1) onRate3Dka=1
> - A(a)+A(b)<-> A(a!1).A(b!1) onRate3Dka=2
>
> The rate for the true self interaction (i.e. onRate3Dka=1 in the example above), is the one that corresponds to the $K_D$ given all identical sites. If this change is not done by the user, and the same rates [illegible]

In addition, the choice of a pre-factor of 20 is because the default clathrin-clathrin binding rate usually results in a single cluster (Figure S5 in the revision), while the experimental data in new Figure S1D show the presence of multiple clusters within a 1 μm × 1 μm square. Since the clathrin-

clathrin binding rate in [11] was estimated by fitting to clathrin dynamics in a membrane tube, the actual clathrin-clathrin binding rate in real cells may differ from that in [11]. So, to explore the mechanism of formation of multiple clusters observed in real cells, we artificially increased the binding rate and examined its effect on cluster formation.

**Actions taken:** To correct the value of default clathrin-clathrin binding rate with AP-2, in the revision, we changed all 1.83 $\mu M^{-1}s^{-1}$ to 0.913 $\mu M^{-1}s^{-1}$, which is the same as that used in ref. 30.

To demonstrate why reducing the clathrin–clathrin binding rate when clathrin is not bound to AP-2 from 0.083 $\mu M^{-1}s^{-1}$ to 0, we added a new Figure S2 and added the following text on lines 122-128: Moreover, to achieve rapid clathrin-clathrin binding near the membrane, the binding rate is modeled with two distinct values depending on whether at least one clathrin is bound to AP-2: the rate is set to zero if neither interacting clathrin is bound to AP-2, and non-zero if at least one of them is bound to AP-2. Note that, we set the clathrin–clathrin binding rate to zero (instead of the 0.083 $\mu M^{-1}s^{-1}$ reported in [30]) when neither clathrin is bound to AP-2, as this results in a higher number of clusters compared to a non-zero binding rate (Figure S2).

Next, to explain why we multiply 0.913 $\mu M^{-1}s^{-1}$ by 20 as the clathrin-clathrin binding rate when at least one clathrin is bound to AP-2, we added a new Figure S1D and the text on lines 279-281 and 293-296. In details, the new Figure S1D shows the presence of multiple clusters within a 1 μm×1 μm square area, which cannot be observed when directly simulating clathrin dynamics with the clathrin-clathrin binding rate from ref. 30 (Figure S5). The text explains that the binding rate used in ref. 30 may not be suitable for real cells.

*5. In sections 3.4 and 3.5 the authors vary the diffusion coefficient of clathrin, reducing it by as much as a factor 40. What is the physical motivation for this variation? Does the triskelion change shape or does the cytosol viscosity change when a cell is exposed to EGF? Since the diffusion coefficient of a membrane-bound lattice is dominated by the number of membrane anchors, an increase in the number of AP2s would be a more natural explanation of a reduced lattice diffusion coefficient.*

**Response:** We thank the reviewer for the good suggestion. In Section 3.4, we reduced the clathrin diffusion coefficient primarily because the value used in previous sections may be higher than those reported for real cells, as indicated in [12, 13]. Specifically, the diffusion coefficient of clathrin light chain is reported to be 0.0096 $\mu m^2 s^{-1}$ in [12], while clathrin on the nuclear surface shows a diffusion coefficient of 0.25 $\mu m^2 s^{-1}$ [13]. Compared with the values reported in the two literature above, the higher diffusion coefficient used in the previous sections may result from the assumption that the surrounding environment of clathrin is water, which is less viscous than the cytosol or membrane.

In section 3.5, as EGF triggers the aggregation of various molecules, including $\beta 5$ integrin and EGF receptors, into the clathrin cluster [10], the clathrin diffusion coefficient ($D_{Clat}$) may decrease in response to the EGF stimulus. This is why we reduce the value of the clathrin diffusion coefficient, $D_{Clat}$.

**Actions taken:** In the revision, to clarify the rationale behind reducing the clathrin diffusion coefficient in sections 3.4 and 3.5, we added the text on lines 316-320 and 341-344, which is also reproduced here:

(1) in the section 3.4: "In the above analysis, we assumed that the surrounding environment of clathrin is water, which is less viscous than the cytosol. Therefore, the diffusion coefficient of clathrin in real cells [58,59] is usually smaller than the value used in the analysis. To investigate how the diffusion coefficient of clathrin affects the formation of multiple clusters, we next varied

the clathrin diffusion coefficient while keeping other kinetic parameters constant."

(2) in the section 3.5: "The increase in clathrin-clathrin binding rate may result from EGF-induced phosphorylation of the clathrin heavy chain [60], while the decrease in $D_{Clat}$ may be attributed to EGF-triggered aggregation of various molecules, including β5 integrin and EGF receptors, into the clathrin cluster [16]."

## Minor comments:

** The abbreviation FCL is used in the abstract without proper introduction.*

**Response:** We thank the reviewer for pointing this out.

**Actions taken:** In the revision, we added the abbreviation "FCL" immediately after we first mentioned flat clathrin lattice in the Abstract.

** Since reference 30 contains the original simulation model, I googled the title – which turned out to be a poster abstract in BPJ. The provided PLOS page number is not correct either; the doi number works.*

**Response:** Sorry for the confusion. The poster abstract in BPJ may indicate that Guo et al. has presented the work in reference 30 in BPJ. The work has now been officially published in PLOS Computational Biology. We have also double-checked the page numbers and volume for reference 30, which are 1–24 and 18, respectively.

**Actions taken:** We have double-checked the citation of reference 30 to ensure its accuracy.

## Reviewer #3 (Comments to the Author):

The authors present a study characterizing the properties that determine the sizes and lifetimes of flat clathrin lattices (FCL) on the membrane, which are known to be important in signalling. They first study how the lattice properties, specifically number, size and dwell time, vary without stimulus. Based on these insights, they then develop time-dependent changes to the model that can explain the FCL response to EGF stimulation. This behavior is an increase in number of FCL clusters, with a simultaneous increase in their size, which they show in the SI is not consistent with a continuum model. Overall, the paper is well- organized and results and figures are clear.

*1. Aside from a few clarifying questions, the paper would be strengthened by a more extensive discussion of why the authors think the increase in FCL number/size is followed by a decrease over 60 minutes back to initial levels? Is the stimulation persistent throughout this whole time, indicating that the cells somehow respond by forming more FCL (or also other types of clathrin-coated structures?) and then return back to 'starting' levels prior to stimulation, despite a persistent addition of EGF? or do they think the return back to starting levels is a relaxation after a brief EGF stimulation (which is then removed)? This might help with the interpretation of the model results, which have to be somewhat artificially forced to reproduce the growth in clusters size/number and its subsequent decay in time.*

**Response:** We thank the reviewer for this suggestion. Since EGF triggers the activation and subsequent deactivation of the EGFR pathway, we assume that the EGFR pathway induces changes in the AP-2 number, clathrin-clathrin binding rate, or clathrin diffusion coefficient between 0 and 60 minutes following EGF stimulation. Moreover, EGFR endocytosis may contribute to the decrease in FCL number and area between 30 and 60 minutes following EGF stimulation. This is because endocytosis requires Ω-shaped pits, which may originate from FCL.

**Actions taken:** To better interpret the condition to achieve the EGF-triggered FCL dynamics, in the revision, we added a new paragraph in the Discussion section on lines 444 to 456, which is also attached here: The way we altered the kinetic parameters in Figure 6B (iii) is closely related to the activity of EGFR pathway. Upon persistent 60-minute EGF stimulation, EGFR and downstream kinases are first activated and then deactivated [67]. At the same time, some downstream molecules also exhibit an enrichment followed by degradation, returning to pre-stimulus levels, for example, Grb2 [68]. Thus, we predict that these changes in EGFR and downstream pathways affect the AP-2 number, clathrin-clathrin binding rate, and clathrin diffusion coefficient, that is, all of these parameters exhibit a sudden change at 30 minutes (as shown in Figure 6B (iii)). Here, the change in AP-2 number can be interpreted as a change in the available AP-2, since the the EGFR pathway may regulate the state of AP-2. In addition, upon EGF stimulation, EGFR endocytosis is triggered on the cell membrane. This may cause the FCL to form Ω-shaped pits that leave from the membrane, leading to a reduction in FCL on the surface. This endocytosis process may contribute to the decrease in FCL number and area between 30 and 60 minutes following EGF stimulation. Further experiments are needed to test these hypotheses.

### Major points:

*2. In Fig3A,it says there are 10 AP2,but >20 clathrin are bound to AP2, how is that possible? Is it including all clathrin that are clathrin bound to the membrane?*

**Response:** Sorry for the confusion. The reviewer is correct, and the appropriate terminology should be "membrane-bound clathrin," rather than "AP-2 bound clathrin." So in Figure 3A, there

are 10 AP-2s but more than 20 clathrin molecules bound to the membrane. The higher number of membrane-associated clathrin compared to AP2 is due to the fact that once a clathrin binds to a membrane-located AP-2, this clathrin molecule can further recruit additional clathrin molecules from the cytosol through clathrin-clathrin interactions. This allows more clathrin to accumulate near the membrane, even without direct binding to AP2 (also see the point 4 from reviewer 2).

**Actions taken:** To clarify this, in the revision, we changed the y label from "Number of AP-2 bound clathrins" to "Number of membrane-bound clathrins" in Figures 3,4,5, and other supplement figures and rewrote the text accordingly.

Moreover, in the revision, we revised the text on lines 122-128 in the Model Development section to better illustrate the underlying mechanism by which clathrin accumulates near the membrane through the interaction between cytosolic clathrin and AP-2 bound clathrin.

*-Also, the snapshots show many many small blue dots—what are those?*

**Response:** Sorry for the confusion. The blue particle represents clathrin in a large membrane-bound cluster (containing more than 10 clathrins). On the contrary, the gray particle represents the clathrin either in the cytosol or in a small membrane-bound cluster (with no more than 10 clathrins).

**Actions taken:** To better show the blue and gray dots, we added zoomed-in insets of these dots in Figures 1F, 2A-B, 3A and added captions for them in the corresponding panels.

*-What does the AP2 look like?*

**Response:** Sorry for the confusion. AP-2 is a zero-volume dot that is located on the cell membrane. The cytosolic clathrin can bind to the membrane-bound AP-2, and thus some some AP-2 molecules are bound to clathrin.

**Actions taken:** In the revision, to clarify how we model the AP-2, we added the following sentence on lines 180-184: As for AP-2, it is represented as a zero-volume dot that is always located on the cell membrane. Although all AP-2 molecules are located on the membrane, they exist in two forms: clathrin-bound and clathrin-unbound. The location of clathrin-bound AP-2 is determined by the clathrin binding site, while clathrin-unbound AP-2 molecules are not explicitly modeled (Figure S3). This implicit localization assumption for AP-2 is based on the *in vitro* model described in [30].

Moreover, to clearly show the locations of AP-2, we added new Figure S3 that highlight the locations of AP-2. In this new figure, red dots represent the clathrin-bound AP-2 molecule. Except for the clathrin-bound AP-2 molecules, the locations of other AP-2 molecules are not shown in the figure. As mentioned above, this is because we model AP-2 molecules implicitly using the NERDSS algorithm [14], which means that the positions of individual AP-2 molecules are not directly tracked throughout the simulation. However, the location of clathrin-bound AP-2 can be inferred from the clathrin binding site.

*3. In Fig4,what copy number of AP2 is being used?It would help to have this readily available in the legend/ text to compare with what was observed in Fig 3.*

**Response:** Thank you for the suggestion. In Figure 4, we set the number of AP-2 to 100 but varied the clathrin-clathrin binding rate. When the clathrin-clathrin binding rate is changed to $50\times0.913$ $\mu M^{-1}s^{-1}$, it corresponds to the third bar in Figure 3D, representing the case with an average of 3 clusters. However, for the system with 100 AP-2 molecules and the clathrin-clathrin binding rate of $50\times0.913$ $\mu M^{-1}s^{-1}$, increasing the number of AP-2 further does not lead to an

increase in cluster number (Figure 3D), but increasing the clathrin-clathrin binding rate significantly increases the cluster number (Figure 4D).

**Actions taken:** In the revision, we added "AP-2 number = 100" in Figure 4 and $k_{(AP\text{-}2\cdot)Clat\cdot Clat} = 50\times0.913\ \mu M^{-1}s^{-1}$ in Figure 3. Moreover, to build the connection of Figures 3 and 4, we added the following text on lines 303-309: "Note that the third bar in Figure 4 is identical to the third bar in Figure 3, as both panels are calculated based on the case where the AP-2 number is 100 and the clathrin-clathrin binding rate is $50\times0.913\ \mu M^{-1}s^{-1}$. For this system, increasing the AP-2 number does not increase the cluster number (fourth to last bars in Figure 3D), while increasing the clathrin-clathrin binding rate significantly increases the cluster number (the last two bars in Figure 4D), indicating that the clathrin-clathrin binding rate has a greater impact on cluster number than increasing the AP-2 number."

*4. How many clathrin are in the simulations? Do they all get recruited to the surface in any simulations, or is there always a reservoir in solution? Is this an important element of the observations—that clathrin is/not in excess of the adaptors?*

**Response:** Thank you for the questions. There are 300 clathrin molecules in total, and this number remains fixed. However, the number of clathrins recruited to the membrane varies depending on the AP-2 number, clathrin-clathrin binding rate, and clathrin diffusion coefficient. For example, when there are 400 AP-2 molecules (Figure 3C), nearly all clathrins are bound to the membrane. Additionally, increasing the clathrin-clathrin binding rate results in more clathrins on the membrane (Figure 4A–C).

**Actions taken:** In the revision, we added the number of clathrins in Table S1. Furthermore, we added the following text in the Discussion section to acknowledge the limitation of our study that the clathrin number is fixed: "In addition, we assumed a fixed number of 300 clathrin molecules, neglecting their production and degradation. As a result, the number of membrane-bound clathrins cannot exceed 300. Given the complexity of the protein synthesis process, we anticipated that clathrin production could influence the results over longer timescales."

*5. The results from changing the diffusion constant are surprising once they have reached what looks like a relatively equilibrated steady-state. At this point, the transport properties, while affecting lifetimes, should not impact the distribution of sampled states if its truly at equilibrium. This type of unexpected behavior was also observed in recent work: Qian et al, https://doi.org/10.1016/j.bpj.2023.06.021 with virus assembly using the NERDSS software (section titled 'Mimicking activation by co- factors.."). That paper identified the issue as related to the coalescence events between intermediate-sized clusters having lower probabilities with slower diffusion. This resembles what you are seeing, with more smaller clusters at slower diffusion. While I agree with the discussion section that notes that slower diffusion when out-of-equilibrium would affect cluster size distribution, it is worth noting this caveat for the steady-state systems. This result could be an artifact of the software that it produces an effectively lower acceptance rate for two clusters to combine with slower diffusion via a phenomenological acceptance criteria.*

**Response:** We thank the reviewer for this thoughtful insight. We agree that although the effect of the diffusion rate on clathrin dynamics reported in the manuscript has also been observed in other studies, it may come from an artifact of the software.

**Actions taken:** To discuss the reliability of the effect of the diffusion rate on clathrin dynamics, in the revision, we added the following text on lines 423-430 in the Discussion section: This type

of behavior was also observed in recent work [61]. However, these results seem to deviate from the property for the steady-state system, where the distribution of clusters is expected to remain unchanged. The possible reason might be that a slower clathrin diffusion coefficient prevents dissociated clathrin from dispersing too far, thus allowing the clathrin clusters to efficiently recruit the dissociated clathrin and maintain their structure, rather than merging into a giant cluster or disassembling. Thus, the effect of the diffusion coefficient on clathrin dynamics could be an artifact of the algorithm, as the phenomenological acceptance criteria can lead to a low acceptance rate for two clusters to combine when molecule diffusion becomes slow.

*6. The comparison to the Turing model is interesting, that although increases in cluster numbers may be common in models, the increase occurs with smaller clusters forming, in opposition to the EGF stimulation response. In the snapshots of Fig S4, the authors show the clathrin distribution. Is it co-localized in this model with the AP2 or anti-localized? One confusing aspect of the comparison is that the AP2/activator is slower than the clathrin/inhibitor. But once clathrin is on the membrane, it diffuses even slower than AP2, it is only faster in solution. Both variables are being tracked on the membrane, as far as I can tell [Clat] and [AP2], meaning they both diffuse slowly. This is one point to note about this model, that if it requires the activator to be slower than the inhibitor, particularly by a large margin, this is not physically justified for AP2 vs clathrin-bound-to-AP2.*

**Response:** Thank you for the question. In the Turing model, the AP-2 is colocalized with the clathrin, as shown in the new Figure S8D. We also agreed that the condition in the Turing model, which requires the activator to diffuse more slowly than the inhibitor, may not be satisfied in the clathrin-AP-2 system.

**Actions taken:** To illustrate the location of AP-2, in the revision, we added a new panel D in Figure S8. By comparing this panel with panel B, we found that AP-2 is co-localized with clathrin.

To demonstrate the non-physical restriction on the diffusion rate required by the Turing model, in the revision, we added the following text on lines 471-475 in the Discussion section: In addition to this shortcoming, the assumption of the Turing model may also be unrealistic for the clathrin-AP-2 system. Specifically, the Turing model requires that AP-2, as an activator, diffuses more slowly than the membrane-bound clathrin. However, this may not hold true in the clathrin-AP-2 system, as the membrane-bound clathrin is a complex composed of both AP-2 and clathrin, which could cause it to diffuse more slowly than free AP-2.

### Minor:

*Last paragraph of discussion has typos, an extraneous 'xt)' for example. This paragraph is also rather hard to read and could be revised.*

**Response:** We thank the reviewer for the suggestions. We have corrected the typo and reorganized the paragraph for clarity.

**Actions taken:** In the revision, we have removed the text "xt)" and other sentences in the last paragraph of the Discussion section to improve its fluency.

## References

[1] Kang, M., Day, C. A., Kenworthy, A. K. & DiBenedetto, E. Simplified equation to extract diffusion coefficients from confocal frap data. *Traffic* **13**, 1589–1600

(2012). URL https://onlinelibrary.wiley.com/doi/abs/10.1111/tra.12008. https://onlinelibrary.wiley.com/doi/pdf/10.1111/tra.12008.

[2] Saffman, P. G. & Delbrück, M. Brownian motion in biological membranes. *Proceedings of the National Academy of Sciences* **72**, 3111–3113 (1975). URL https://www.pnas.org/doi/abs/10.1073/pnas.72.8.3111. https://www.pnas.org/doi/pdf/10.1073/pnas.72.8.3111.

[3] Gambin, Y. *et al.* Lateral mobility of proteins in liquid membranes revisited. *Proceedings of the National Academy of Sciences* **103**, 2098–2102 (2006). URL https://www.pnas.org/doi/abs/10.1073/pnas.0511026103. https://www.pnas.org/doi/pdf/10.1073/pnas.0511026103.

[4] Pincet, F. *et al.* Frap to characterize molecular diffusion and interaction in various membrane environments. *PLOS ONE* **11**, 1–19 (2016). URL https://doi.org/10.1371/journal.pone.0158457.

[5] Richbourg, N. R. & Peppas, N. A. High-throughput frap analysis of solute diffusion in hydrogels. *Macromolecules* **54**, 10477–10486 (2021). URL https://doi.org/10.1021/acs.macromol.1c01752.

[6] Alexander, A. M. & Lawley, S. D. Inferences from frap data are model dependent: A subdiffusive analysis. *Biophysical Journal* **121**, 3795–3810 (2022). URL https://doi.org/10.1016/j.bpj.2022.09.015.

[7] Hubatsch, L. *et al.* Quantitative theory for the diffusive dynamics of liquid condensates. *eLife* **10**, e68620 (2021). URL https://doi.org/10.7554/eLife.68620.

[8] Sochacki, K. A. *et al.* The structure and spontaneous curvature of clathrin lattices at the plasma membrane. *Developmental Cell* **56**, 1131–1146.e3 (2021). URL https://doi.org/10.1016/j.devcel.2021.03.017.

[9] Grove, J. *et al.* Flat clathrin lattices: stable features of the plasma membrane. *Molecular Biology of the Cell* **25**, 3581–3594 (2014). URL https://doi.org/10.1091/mbc.e14-06-1154. PMID: 25165141, https://doi.org/10.1091/mbc.e14-06-1154.

[10] Alfonzo-Méndez, M. A., Sochacki, K. A., Strub, M.-P. & Taraska, J. W. Dual clathrin and integrin signaling systems regulate growth factor receptor activation. *Nature Communications* **13**, 905 (2022). URL https://doi.org/10.1038/s41467-022-28373-x.

[11] Guo, S.-K., Sodt, A. J. & Johnson, M. E. Large self-assembled clathrin lattices spontaneously disassemble without sufficient adaptor proteins. *PLOS Computational Biology* **18**, 1–24 (2022). URL https://doi.org/10.1371/journal.pcbi.1009969.

[12] Pelassa, I., Zhao, C., Pasche, M., Odermatt, B. & Lagnado, L. Synaptic vesicles are "primed" for fast clathrin-mediated endocytosis at the ribbon synapse. *Frontiers in Molecular Neuroscience* **Volume 7 - 2014** (2014). URL https://www.frontiersin.org/journals/molecular-neuroscience/articles/10.3389/fnmol.2014.00091.

[13] Nandi, P. & Wahl, P. Diffusion properties of clathrin on the surface of isolated mouse liver nuclei by the fluorescence recovery after photobleaching technique **943**, 367–370 (1988). URL https://www.sciencedirect.com/science/article/pii/000527368890569X.

[14] Varga, M. J., Fu, Y., Loggia, S., Yogurtcu, O. N. & Johnson, M. E. Nerdss: A nonequilibrium simulator for multibody self-assembly at the cellular scale. *Biophysical Journal* **118**, 3026–3040 (2020). URL https://www.sciencedirect.com/science/article/pii/S0006349520303970.
