# Supplementary material for: Dynamics of the formation of flat clathrin lattices in response to growth factor stimulus
Source: PLoS Comput Biol. 2026 Mar 11;22(3):e1014013. doi: 10.1371/journal.pcbi.1014013 (PMC13012621; doi:10.1371/journal.pcbi.1014013)
Supplement: Attachment — Submitted filename: Referee report for Qiao et al.pdf [file pcbi.1014013.s015.pdf]

Referee report for Qiao et al.

The authors present a study characterizing the properties that determine the sizes and lifetimes of flat clathrin lattices (FCL) on the membrane, which are known to be important in signalling. They first study how the lattice properties, specifically number, size and dwell time, vary without stimulus. Based on these insights, they then develop time-dependent changes to the model that can explain the FCL response to EGF stimulation. This behavior is an increase in number of FCL clusters, with a simultaneous increase in their size, which they show in the SI is not consistent with a continuum model. Overall, the paper is well-organized and results and figures are clear. Aside from a few clarifying questions, the paper would be strengthened by a more extensive discussion of why the authors think the increase in FCL number/size is followed by a decrease over ~60 minutes back to initial levels? Is the stimulation persistent throughout this whole time, indicating that the cells somehow respond by forming more FCL (or also other types of clathrin-coated structures?) and then return back to 'starting' levels prior to stimulation, despite a persistent addition of EGF? or do they think the return back to starting levels is a relaxation after a brief EGF stimulation (which is then removed)? This might help with the interpretation of the model results, which have to be somewhat artificially forced to reproduce the growth in clusters size/number and its subsequent decay in time.

Major points:

1. In Fig 3A, it says there are 10 AP2, but >20 clathrin are bound to AP2, how is that possible? Is it including all clathrin that are clathrin bound to the membrane? Also, the snapshots show many many small blue dots—what are those? What does the AP2 look like?
2. In Fig 4, what copy number of AP2 is being used? It would help to have this readily available in the legend/ text to compare with what was observed in Fig 3.
3. How many clathrin are in the simulations? Do they all get recruited to the surface in any simulations, or is there always a reservoir in solution? Is this an important element of the observations—that clathrin is/not in excess of the adaptors?
4. The results from changing the diffusion constant are surprising once they have reached what looks like a relatively equilibrated steady-state. At this point, the transport properties, while affecting lifetimes, should not impact the distribution of sampled states if its truly at equilibrium. This type of unexpected behavior was also observed in recent work: Qian et al, https://doi.org/10.1016/j.bpj.2023.06.021 with virus assembly using the NERDSS software (section titled 'Mimicking activation by co-factors..”). That paper identified the issue as related to the coalescence events between intermediate-sized clusters having lower probabilities with slower

diffusion. This resembles what you are seeing, with more smaller clusters at slower diffusion. While I agree with the discussion section that notes that slower diffusion when out-of-equilibrium would affect cluster size distribution, it is worth noting this caveat for the steady-state systems. This result could be an artifact of the software that it produces an effectively lower acceptance rate for two clusters to combine with slower diffusion via a phenomenological acceptance criteria.

5. The comparison to the Turing model is interesting, that although increases in cluster numbers may be common in models, the increase occurs with smaller clusters forming, in opposition to the EGF stimulation response. In the snapshots of Fig S4, the authors show the clathrin distribution. Is it colocalized in this model with the AP2 or anti-localized? One confusing aspect of the comparison is that the AP2/activator is slower than the clathrin/inhibitor. But once clathrin is on the membrane, it diffuses even slower than AP2, it is only faster in solution. Both variables are being tracked on the membrane, as far as I can tell [Clat] and [AP2], meaning they both diffuse slowly. This is one point to note about this model, that if it requires the activator to be slower than the inhibitor, particularly by a large margin, this is not physically justified for AP2 vs clathrin-bound-to-AP2.

Minor:

Last paragraph of discussion has typos, an extraneous ‘xt)’ for example. This paragraph is also rather hard to read and could be revised.
